# Integrated RNA-seq and sQTL analysis reveal immune and splicing regulatory features underlying relapse and remission after treatment of Graves’ disease

**DOI:** 10.3389/fendo.2026.1791850

**Published:** 2026-05-13

**Authors:** Yang Wu, Jie Liu, Yu Yang, Kun Chen, Fei Hua, Kun Wang

**Affiliations:** 1 Department of Endocrinology, The Third Affiliated Hospital of Soochow University, Changzhou, Jiangsu, China; 2 Department of Endocrinology, The Affiliated Jiangning Hospital of Nanjing Medical University, Nanjing, Jiangsu, China

**Keywords:** AS, Graves’ disease, RBP, RNA-Seq, SQTL

## Abstract

**Introduction:**

Graves' disease (GD) is a classical autoimmune disorder caused by interactions between genetic susceptibility and immune dysregulation. However, the transcriptomic mechanisms underlying disease relapse and remission, particularly those involving alternative splicing (AS), remain poorly understood.

**Methods:**

We performed an integrative RNA-seq analysis of peripheral blood samples from 33 GD patients in remission, 31 GD patients in relapse, and 30 normal controls (NC). Gene expression, transcript usage, alternative splicing events (ASEs), RNA-binding protein (RBP) regulation, and splicing quantitative trait loci (sQTLs) were systematically analyzed to characterize transcriptomic alterations associated with different disease states.

**Results:**

Compared with NC, relapse-associated differentially expressed genes (DEGs) were mainly enriched in antimicrobial humoral immunity and suppression of TNF signaling, whereas remission-associated DEGs were associated with metabolic homeostasis and apoptosis regulation. Notably, 74 DEGs were consistently upregulated in both disease states. Transcriptomic analysis identified 234,595 transcripts, including 17.1% novel isoforms, and detected 352 and 387 differentially expressed transcripts (DETs) in relapse and remission, respectively. Transcript-level alterations frequently occurred independently of gene-level expression changes, indicating extensive isoform-specific regulation, as exemplified by isoform switching of *HELZ2*. In addition, 858 relapse-associated and 670 remission-associated aberrant ASEs (AASEs) were identified. RBP-AASE regulatory network analysis suggested that key RBPs, including *APOBEC3C*, may contribute to stage-specific splicing remodeling. sQTL analysis further identified 4,507 significant SNP-ASE associations, with affected genes enriched in immune-related pathways, including Th1/Th2/Th17 cell differentiation and TNF signaling. Among these, 12 splicing-related genes also exhibited aberrant AS patterns, while *DDX5* and *PKM* showed sustained upregulation in both relapse and remission phases.

**Discussion:**

These findings demonstrate that GD relapse and remission are closely associated with genetically and RBP-mediated alternative splicing regulation. Our study provides new insights into the molecular mechanisms underlying immune imbalance in GD and highlights potential transcriptomic biomarkers associated with disease relapse.

## 1 Introduction

Graves’ disease (GD), also known as toxic diffuse goiter, is a chronic autoimmune thyroid disorder that affects multiple organ systems (1). GD is the most common cause of hyperthyroidism, accounting for approximately 80% of all hyperthyroid cases and affecting about 1% of the general population (2, 3). The clinical manifestations of GD mainly include hypermetabolic syndrome, diffuse goiter, pretibial myxedema, and ophthalmopathy (4). Although thyroidectomy can rapidly alleviate hyperthyroid symptoms, patients require lifelong thyroid hormone replacement therapy, and the procedure itself may lead to various complications, such as hypoparathyroidism (5). Therefore, an in-depth understanding of the molecular pathogenesis of GD, as well as its genetic and molecular regulatory mechanisms, is of great importance for identifying high-risk populations and optimizing personalized treatment strategies.

The rapid development of high-throughput RNA sequencing (RNA-seq) technologies has provided powerful tools for systematically characterizing disease-related transcriptomic landscapes in humans (6). RNA-seq enables the simultaneous acquisition of genome-wide expression profiles at both the gene and transcript levels, allowing for the identification of differentially expressed genes (DEGs) and differentially expressed transcripts (DETs), which can then be linked to immune cell composition, signaling pathway activity, and clinical phenotypes (7). Accumulating evidence suggests that systematic analysis of DEGs and DETs facilitate the identification of aberrantly activated or suppressed immune pathways in autoimmune diseases, and help uncover key molecules associated with disease activity, prognosis, and therapeutic response, thereby providing important clues for the discovery of novel biomarkers and potential therapeutic targets (8, 9). For example, Zhang et al. constructed a prognostic model based on DEG signatures from peripheral blood samples of patients with lung cancer and healthy controls, and identified four risk factors, *CEACAM6*, *CEACAM1*, *HK3*, and *SLC36A1*, that were highly expressed in lung cancer samples (10). Liu et al. identified aberrantly expressed lncRNAs in peripheral blood from patients with GD compared with normal controls and found that *ENST00000604491* was significantly downregulated in GD patients, participating in GD pathogenesis through the regulation of *FOXP1* (11). Notably, while comparisons with healthy controls primarily capture disease-associated alterations, direct comparison between post-treatment remission and relapse can more precisely delineate molecular programs underlying disease activity dynamics. Such analyses help uncover regulatory pathways and immune processes specifically linked to disease reactivation or sustained remission, thereby providing insights into mechanisms of treatment response and failure. Importantly, these stage-specific transcriptomic features may offer clinically actionable information for relapse prediction, disease monitoring, and individualized therapeutic decision-making that cannot be readily inferred from case–control comparisons alone. However, comparative transcriptomic studies of peripheral blood focusing on GD, particularly across the two critical clinical stages of post-treatment remission and post-treatment relapse, remain relatively limited. The fine-scale regulatory features at the transcript level during these stages, as well as their relationships with immune dysregulation, have yet to be fully elucidated.

Alternative splicing (AS) is one of the core mechanisms determining transcript diversity and the functional complexity of proteins, with more than 90% of human multi-exon genes undergoing AS events (ASEs) (6). Substantial evidence has demonstrated that dysregulation of AS is broadly involved in the initiation and progression of a wide range of human diseases, including cancers, neurological disorders, and immune-related diseases (12, 13). In this process, RNA-binding proteins (RBPs) act as key splicing regulators, exerting fine control over splice-site selection and isoform balance; aberrant expression or dysfunction of RBPs can drive disease-associated splicing programs (14). On the other hand, analysis of expression quantitative trait loci (eQTLs) and splicing quantitative trait loci (sQTLs), which link genomic genetic variants to expression or splicing phenotypes, have become important approaches for elucidating the causal relationships between genetic susceptibility and post-transcriptional regulation in complex diseases (15). eQTL/sQTL analysis establish a “functional bridge” between individual genetic backgrounds, gene expression/splicing patterns, and disease risk, providing new perspectives for understanding the genetic architecture of complex autoimmune diseases (16, 17). Increasing evidence indicates that sQTL analysis can precisely pinpoint pathogenic splicing variants and functionally susceptible genes across diverse complex diseases. To date, numerous disease-associated genes have been identified through sQTL mapping, including *LAG3* in autoimmune thyroid diseases, *L3MBTL3* in multiple sclerosis, *TCHP* and *ZNF76* in systemic lupus erythematosus, and *FARP1* in non-small cell lung cancer (18–22). However, systematic investigations of eQTL or sQTL regulatory patterns in GD—particularly those related to disease relapse—are still lacking.

In this study, we performed RNA-seq analysis on healthy controls (NC) as well as post-treatment remission and post-treatment relapse groups of GD patients to systematically characterize the transcriptomic features of GD. We annotated and classified 234,595 transcripts identified from the RNA-seq data and compared expression differences between remission and relapse states at both the gene and transcript levels. Furthermore, we systematically identified AASEs in the remission and relapse states and constructed RBP–AASE splicing relevance networks specific to each condition. In addition, sQTL analysis identified two susceptibility genes, *DDX5* and *PKM*, whose expression levels were significantly elevated in peripheral blood samples from GD patients. Collectively, these results provide a comprehensive depiction of transcriptomic differences in GD across multiple layers, including differential gene and transcript expression, AS, RBP-mediated regulation, and genetic regulation, thereby offering potential targets and new insights for future therapeutic strategies in GD.

## 2 Materials and methods

### 2.1 Sample collected

Peripheral blood samples of 33 GD patients in the post-treatment remission group (remission), 31 GD patients in the post-treatment relapse group (relapse), and 30 age-matched adult healthy controls (NC) were used in this study (**Supplementary Table 1**). All of the samples were recruited from the Jiangning Hospital Affiliated to Nanjing Medical University. The diagnosis of GD was based on characteristic clinical manifestations and abnormal biochemical indicators. Patients with recurrent hyperthyroidism were treated with methimazole tablets (approval number: H20205041), with doses ranging from 5 mg to 30 mg. Healthy controls had no history of autoimmune diseases, tumors, allergic disorders, infectious diseases, or acute or chronic visceral diseases. The GD Remission group consisted of patients who remained euthyroid (TSH: 0.75–5.60 mIU/L; FT3: 3.85–6.30 pmol/L; FT4: 12.80–21.30 pmol/L) for over one year after standardized anti-thyroid drug (ATD) withdrawal. The GD Relapse group, showed clinical recurrence confirmed by FT3 > 6.30 pmol/L, FT4 > 21.30 pmol/L, TSH < 0.75 mIU/L, and TRAb > 1.75 IU/L. Peripheral blood samples from both patients and controls were collected using EDTA-K2 anticoagulant tubes for subsequent transcriptome sequencing.

### 2.2 RNA extraction, library construction, and sequencing

Peripheral blood samples were collected and immediately processed for total RNA extraction. Briefly, total RNA was isolated from peripheral blood using total RNA extraction was obtained using Gene All Hybrid-RTM blood RNA extraction kit (catno.305101) according to the manufacturer’s instructions. RNA concentration and purity were assessed using a spectrophotometer, and RNA integrity was evaluated using an automated electrophoresis system. Only samples with an absorbance wavelength ratio (A260/A280) ≥ 1.9 and an RNA integrity number ≥ 8 were used for subsequent library construction, and samples with borderline or lower RIN values were excluded from further analysis.

For library preparation, a defined amount of high-quality total RNA was used as input. Ribosomal RNA was removed, and the remaining RNA was fragmented into short fragments, followed by first- and second-strand cDNA synthesis. After end repair, A-tailing, and adaptor ligation, cDNA fragments of appropriate size were selected and amplified by PCR to generate the final sequencing libraries. Library quality and concentration were assessed prior to sequencing. The qualified libraries were sequenced on an Illumina sequencing platform, generating paired-end reads with a read length of 150 bp.

### 2.3 Transcript identification

Raw data were subjected to quality control using fastp (v0.23.4) (23) with default parameters to remove adapters and low-quality reads. Subsequently, the clean reads were aligned to the human reference genome GRCh38.p14 (https://www.gencodegenes.org/human/) using HISAT2 (v2.2.1) (24) with default parameters. The resulting BAM files were sorted and indexed using the sort and index functions of SAMtools (v1.19.2) (25). To obtain comprehensive transcript information, transcript assembly was performed based on the sorted BAM files and the GRCh38.p14 genome annotation using StringTie (v1.3.6) (26), with a minimum transcript TPM threshold of 0.1 (-T 0.1) applied to exclude lowly expressed transcripts. The transcript assemblies from all samples were then combined using the StringTie merge function. The merged transcripts were compared against the reference genome annotation using GffCompare (v0.11.2) (27) with default parameters to filter out antisense and fusion transcripts. Furthermore, non-canonical and redundant transcripts were removed using gffread (v0.12.7) (27) with the parameters -N -M -K. Regarding newly identified transcripts, only those located within reference genes or intergenic regions were retained. Finally, the protein-coding potential of the transcripts was predicted using TransDecoder (v.5.5.0, https://github.com/TransDecoder/), and transcripts containing at least one open reading frame (ORF) were retained.

### 2.4 Quantification of genes and transcripts and differential expression analysis

Transcript expression levels in each sample were quantified using Salmon (v1.4.0) (28) with the --gcBias and --seqBias parameters, and abundance was expressed as Transcripts Per Kilobase per Million mapped reads (TPM). Subsequently, read counts and TPM quantification results for all transcripts across all samples were aggregated using the quantmerge module in Salmon. Transcript-level quantification data were summarized into a gene-level read count matrix using the R package tximport (v1.30.0) (29). Differentially expressed genes (DEGs) and differentially expressed transcripts (DETs) were identified in two comparison groups (relapse vs. NC and remission vs. NC) using the R package DESeq2 (v1.42.0) (30). Genes and transcripts meeting the criteria of adjusted P-value (padj) < 0.05 and |log_2_FoldChange| ≥ 1 were defined as DEGs and DETs, respectively.

### 2.5 Identification of AASEs

ASEs were characterized using the generateEvents module of SUPPA2 (v2.3) (31) with the parameters “-e SE SS MX RI FL -f ioe”. Subsequently, the percent spliced-in (PSI) values for each ASE across all samples were quantified using the psiPerEvent module. To ensure the reliability of downstream analysis, we retained ASEs exhibiting a PSI value ≥ 0.1 in at least 5% of the samples. Differential splicing analysis was performed between the comparison groups (relapse vs. NC and remission vs. NC) using the diffSplice module with the parameters “--nan-threshold 0.8, -m classical, and -gc”. Finally, AASEs were identified based on the significance thresholds of p-value < 0.05 and |Delta PSI| > 0.1.

### 2.6 Construction of the regulatory splicing network between RBPs and AASEs

A total of 1,236 human RBPs were compiled through a systematic literature review (**Supplementary Table 2**) (32). To investigate the potential regulatory relationships between RBPs and AASEs in GD, we performed pairwise correlation analysis between all differentially expressed RBPs and AASEs. The potential influence of RBP expression variations on AASE splicing patterns was evaluated by calculating the Spearman correlation coefficient (SCC) between RBP expression levels and the PSI values of AASEs. Multiple testing correction was applied to P-values using the Benjamini–Hochberg method. Following established criteria (33), RBP-AASE associations were considered statistically significant if they met the thresholds of |SCC| > 0.5 and p.adjust < 0.05. Finally, the RBP-AASE splicing regulatory network was visualized using Cytoscape (34).

### 2.7 Identification of SNPs

SNPs were identified across 94 RNA-seq samples following the Calling Variants in RNAseq pipeline (https://github.com/broadgsa/gatk/blob/master/doc_archive/methods/Calling_variants_in_RNAseq.md. Initially, RNA-seq reads were aligned to the reference genome using STAR (v2.7.10a) (35) in “—twopassMode” Basic mode. Read group information was added to the resulting BAM files using the AddOrReplaceReadGroups tool within GATK (36). Duplicate reads were identified and marked using MarkDuplicates to minimize technical bias, followed by sorting with SAMtools. To account for the spliced nature of RNA-seq data, the SplitNCigarReads tool was employed to partition reads into exon segments (removing Ns while maintaining grouping information) and to hard-clip sequences overhanging into intronic regions. Variant calling was performed using GATK HaplotypeCaller with the --dont-use-soft-clipped-bases parameter to prevent interference from soft-clipped bases, and a minimum confidence threshold was established using -stand-call-conf 20.0.

To achieve a more comprehensive set of SNP genotypes, missing data were imputed using Beagle (v5.5) (37) guided by phased VCF reference files obtained from the International Genome Sample Resource (IGSR, https://www.internationalgenome.org/data-portal/data-collection/30x-grch38). The imputed SNPs were refined using GATK VariantFiltration with the parameters “-window 35” and “-cluster 3” to enhance sensitivity and specificity while minimizing false positives. Subsequently, only biallelic SNPs were retained using GATK SelectVariants with the “--restrict-alleles-to BIALLELIC” option. Finally, VCFtools (v0.1.16) (38) was utilized to exclude SNPs with a missing rate exceeding 30% (--max-missing 0.7) or a minor allele frequency (MAF) less than 1% (--maf 0.01).

### 2.8 Identification of sQTLs

To elucidate the potential impact of genetic variants on alternative splicing regulation, we performed an sQTL analysis. Initially, to minimize background noise and exclude low-confidence splicing events, only ASEs with a PSI value > 0 in more than 70% of the samples were retained as candidates for downstream analysis. Population structure was accounted for by performing principal component analysis (PCA) on the genotype data using the R package SNPRelate (v1.44) (39). The first three principal components (PCs) were extracted and incorporated as covariates in the sQTL mapping model. We identified sQTLs significantly associated with PSI values using FastQTL (v2.0) (39). Consistent with established methodologies (22, 40, 41), sQTLs located within a 1 Mb window upstream or downstream of the alternative splicing site were defined as cis-sQTLs. Multiple testing correction was performed using the Benjamini-Hochberg method, and a significance threshold of adjusted P-value (p.adjust) < 0.05 was applied to identify significant sQTL associations. Manhattan plots were generated using the R package CMplot (v4.5.1) (42) to visualize the distribution of SNPs across the genome and their corresponding significant association signals.

### 2.9 Functional enrichment analysis

Gene functional annotations were performed using Eggnog-mapper (43), KOBAS (44), and HMMER (45) with default parameters to obtain Gene Ontology (GO) terms, Kyoto Encyclopedia of Genes and Genomes (KEGG) pathways, respectively. Functional enrichment analysis were conducted using the R package clusterProfiler (v4.0) (46). A significance threshold of adjusted P-value (p.adjust < 0.05), calculated using the Benjamini–Hochberg method, was applied to identify significantly enriched terms and pathways. Finally, the enrichment results were visualized using the R package ggplot2 (v3.5.1) (47).

## 3 Results

### 3.1 Differential gene expression analysis in GD

**Figure 1:**
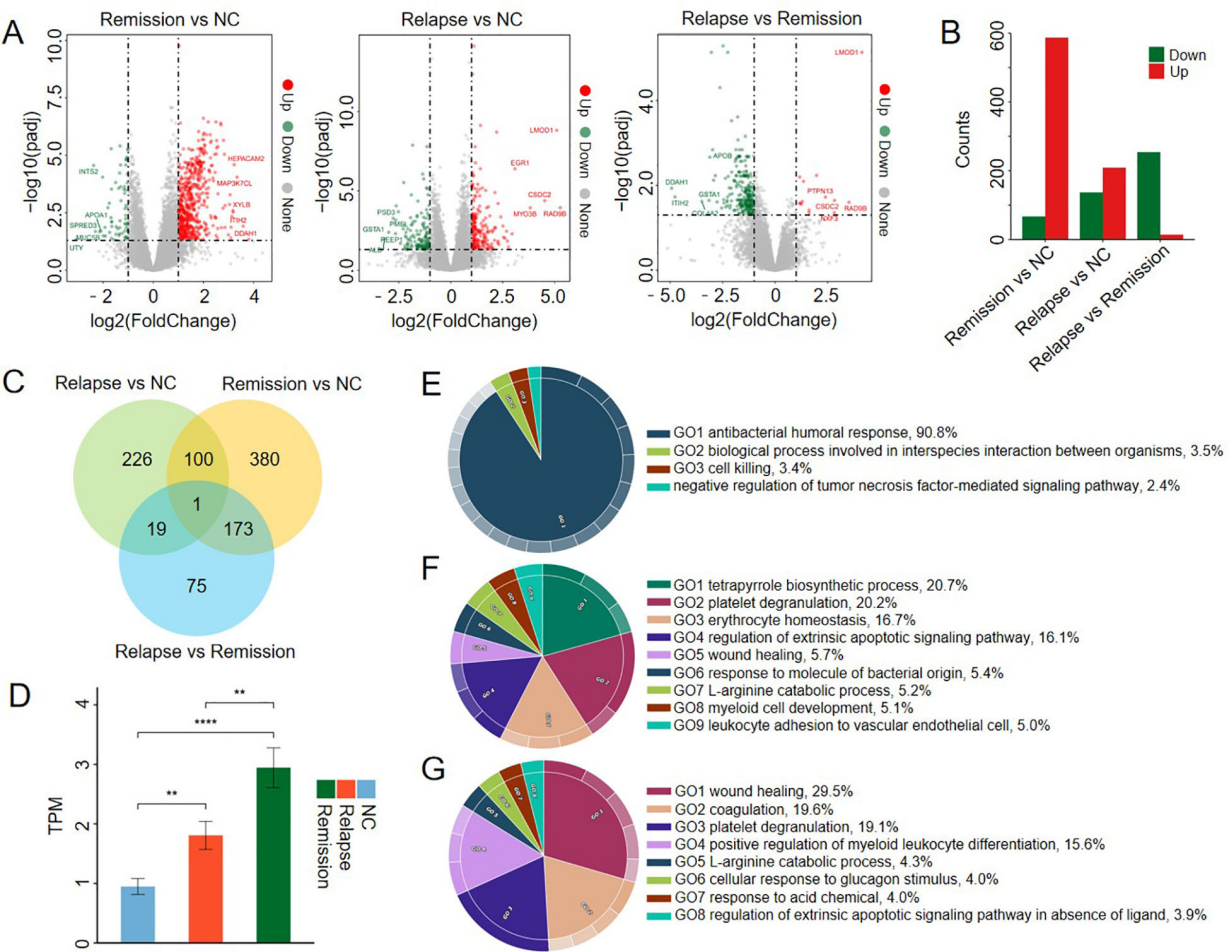
Identification and functional enrichment analysis of DEGs in the Relapse and the Remission groups. **(A)** Volcano plots illustrating differential gene expression in Relapse vs. NC, Remission vs. NC, and Relapse vs. Remission comparison groups (padj < 0.05 and |log2FoldChange| ≥ 1). Red dots represent significantly up-regulated genes, green dots represent significantly down-regulated genes, and gray dots indicate genes with no significant change in expression. In addition, we identified the five upregulated and downregulated genes with the largest fold changes. **(B)** Bar chart displaying the number of up-regulated and down-regulated DEGs in the Relapse vs. NC, Remission vs. NC, and Relapse vs. Remission comparison groups. **(C)** Venn diagram showing the overlap of DEGs between the Relapse vs. NC, Remission vs. NC comparisons, and Relapse vs. Remission comparison groups. **(D)** The bar chart shows the expression levels of the HELZ2 gene and its specific isotypes in the NC, normal control, relapse, and remission groups. The height of the bar represents the mean, and the error bar represents the standard error. Independent samples t-tests (student’s t-test) were used to compare differences among the relapse, remission, and normal control groups. “**” indicates a statistically significant difference (P < 0.01). **(E)** Circos plot representing the functional enrichment results of down-regulated DEGs in the Relapse vs. NC group. **(F)** Circos plots illustrating the functional enrichment of up-regulated DEGs in the Remission vs. NC group. **(G)** Circos plots illustrating the functional enrichment of up-regulated DEGs in the Relapse vs. Remission group.

To dissect the molecular architecture underlying the transition between clinical states, we systematically analyzed gene expression profiles across three comparison groups (Relapse vs. NC, Remission vs. NC, and Relapse vs. Remission) based on transcriptome sequencing data from 94 peripheral blood samples (**Figure 1A**; **Supplementary Figure 1**). Compared with the NC group, a total of 346 DEGs were identified in the Relapse group, comprising 209 up-regulated and 137 down-regulated genes. In the Remission group, 654 DEGs were identified, including 587 up-regulated and 67 down-regulated genes. In the Relapse vs. Remission group, 268 DEGs were identified, including 14 up-regulated and 254 down-regulated genes (**Figure 1B**). Venn diagram analysis revealed that 226, 380 and 75 DEGs were unique to the Relapse vs. NC, Remission vs. NC, and Relapse vs. Remission comparison groups, respectively, while just 1 DEG (*EPB42*) were shared between them (**Figure 1C**). The expression levels of the *EPB42* gene significantly varied across the three groups, with the Remission group showing the highest expression, which was significantly greater than both the Relapse group and the NC group (**Figure 1D**). This suggests that elevated *EPB42* expression may be a key molecular indicator associated with disease remission. This observation suggests that clinically euthyroid remission does not equate to a complete normalization of the transcriptome. Instead, a sustained transcriptional perturbation remains established. Specific down-regulation of cell cycle regulators such as *CDC25C* implies a latent alteration in immune cell proliferation dynamics. These shared DEGs likely represent the intrinsic pathogenic signature of GD that predisposes patients to recurrence, challenging the concept of remission as a complete immunological recovery.

Unraveling the functional architecture of these transcriptional perturbations revealed a fundamental asymmetry in disease regulation. In the Relapse group, the up-regulated gene set lacked convergence on specific biological pathways, suggesting that the pro-inflammatory burst is likely driven by functionally diverse activation or stochastic transcriptional noise rather than a coordinated program. In stark contrast, the down-regulated signature was highly coherent, showing significant enrichment in the ‘negative regulation of tumor necrosis factor (TNF)-mediated signaling’ and pathways related to humoral immune responses (**Figure 1E**). Mechanistically, the suppression of these negative regulators represents a disinhibition of pro-inflammatory signaling. This implies that disease relapse is precipitated not only by novel triggers, but by a systemic impairment in immune inhibitory checkpoints, leading to the unchecked propagation of TNF-driven autoimmunity (48).

Conversely, the Remission state was characterized by the organized mobilization of homeostatic and reparative programs. Up-regulated DEGs were distinctively enriched in tetrapyrrole biosynthetic processes (linked to mitochondrial bioenergetics, 20.7%), platelet degranulation (20.2%), and critically, the regulation of the extrinsic apoptotic signaling pathway (16.1%) (**Figure 1F**). This profile aligns with the immunomodulatory effects of antithyroid drugs, which promote the apoptotic clearance of autoreactive lymphocytes (48). Furthermore, the upregulation of metabolic pathways indicates that maintaining clinical remission requires an active metabolic reprogramming to support tissue repair and proteostasis. Meanwhile, down-regulated DEGs in the Relapse vs. Remission group were exclusively enriched in wound healing (29.5%), coagulation (19.6%), and platelet degranulation (19.1%) (**Figure 1G**), suggesting a reduced activation of hemostatic and tissue repair processes during relapse compared to remission.

### 3.2 Differential transcripts expression analysis in GD

**Figure 2:**
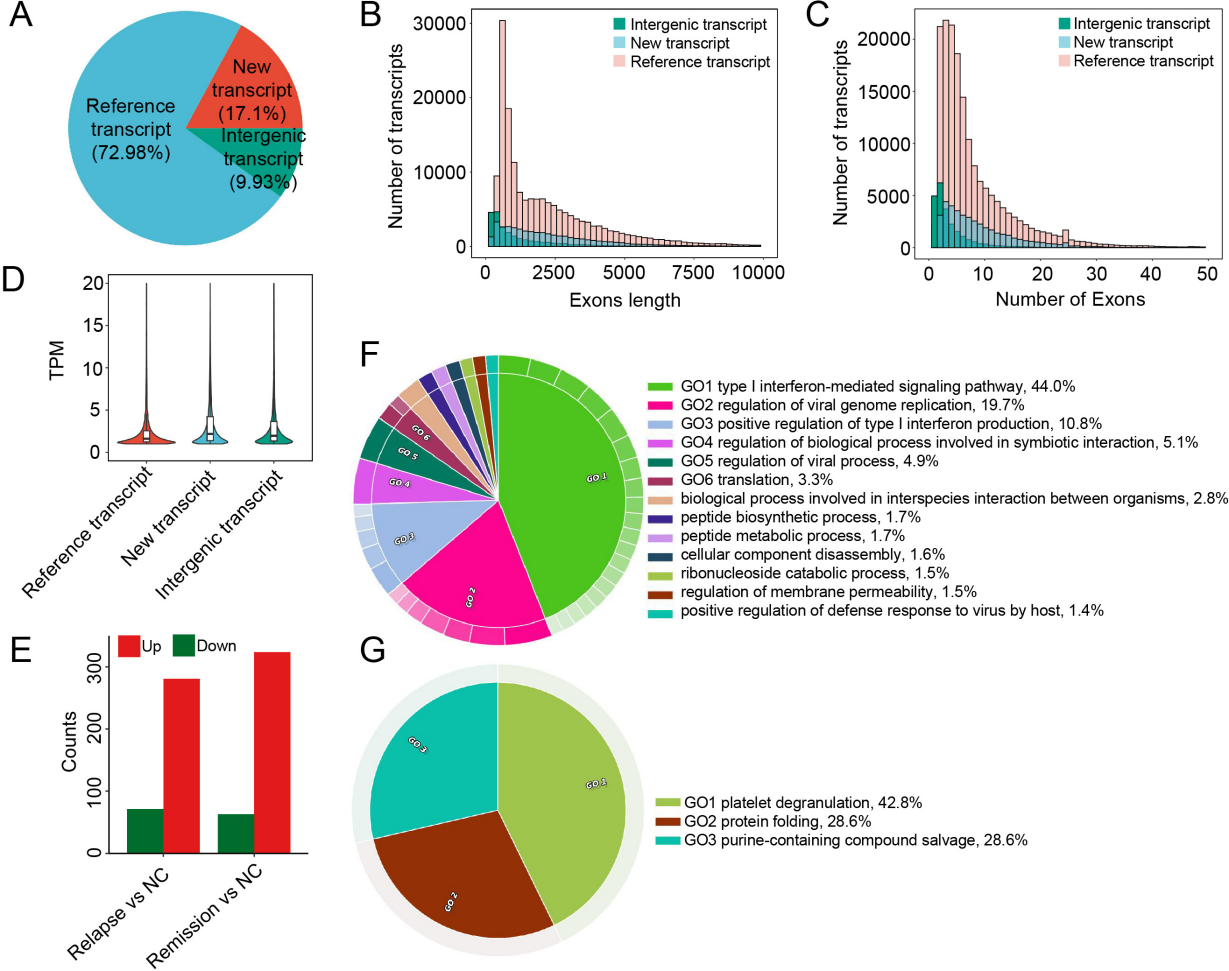
Identification and differential expression analysis of transcripts across 94 RNA-seq samples. **(A)** Pie chart illustrating the proportion of different transcript categories, including reference, novel, and intergenic transcripts. All genes passed the expression threshold of 0.1 TPM. **(B)** Histogram showing the distribution of transcript lengths across the identified transcript types. **(C)** Histogram displaying the distribution of exon counts per transcript. **(D)** Distribution of expression levels (TPM) for the different transcript categories. **(E)** Bar chart showing the number of up-regulated and down-regulated DETs in the Relapse vs. NC and Remission vs. NC comparison groups. **(F, G)** Circos plots illustrating the functional enrichment results of host genes corresponding to DETs in the Relapse vs. NC group **(F)** and the Remission vs. NC group **(G)**.

To further characterize the influence of GD on the human peripheral blood transcriptomic landscape, we performed comprehensive transcript identification across 94 RNA-seq datasets. A total of 234,595 transcripts were identified, comprising 171,198 reference transcripts (73.0%), 40,109 novel transcripts (17.1%), and 23,288 intergenic transcripts (9.9%) (**Figure 2A**). The presence of these newly annotated transcripts reflects the transcriptomic complexity associated with the clinical progression of GD. Analysis of transcript length distribution revealed a gradual decline in the number of transcripts across all three categories as length increased, with the majority of transcripts concentrated within a 5 kb range (**Figure 2B**). Notably, reference transcripts consistently outnumbered novel and intergenic transcripts across all length intervals. Furthermore, analysis of exon numbers demonstrated that transcript frequency decreased as the number of exons increased, with most transcripts containing between 2 and 10 exons (**Figure 2C**). These structural profiles indicate that while reference transcripts are numerically dominant, they also exhibit greater sequence length and higher exon counts, representing more complex structural features. Regarding expression levels, novel and intergenic transcripts showed higher overall expression compared to reference transcripts (**Figure 2D**), suggesting that these newly identified transcripts may play significant roles in GD-related transcriptional regulation.

Subsequently, we systematically identified differentially expressed transcripts (DETs) in the Relapse and Remission groups. A total of 352 DETs were identified in the Relapse group (281 up-regulated and 71 down-regulated), while 387 DETs were identified in the Remission group (324 up-regulated and 63 down-regulated) (**Figure 2E**). To elucidate the potential biological functions of these aberrant transcripts, GO enrichment analysis was performed on their corresponding host genes. The 299 host genes associated with the 352 DETs in the Relapse group were significantly enriched in biological processes closely related to antiviral immune responses, including the type I interferon-mediated signaling pathway (44.0%), regulation of viral genome replication (19.7%), positive regulation of type I interferon production (10.8%), and regulation of viral process (4.9%) (**Figure 2F**). These results indicate that the peripheral blood in the relapse phase of GD is characterized by a significantly activated antiviral immune state. In contrast, the 333 host genes associated with the 387 DETs in the Remission group were primarily enriched in platelet degranulation (42.8%), protein folding (28.6%), and purine-containing compound salvage (28.6%), involving platelet activation, proteostasis maintenance, and nucleotide metabolism. Collectively, these findings reveal distinct transcript-level regulatory patterns between the relapse and remission stages of GD.

### 3.3 Comparative analysis of gene and transcript expression patterns

Alternative splicing of genes can generate multiple transcript isoforms, thereby significantly expanding functional diversity (49). In this study, we conducted a comparative analysis between gene-level and transcript-level expression. In the Relapse group, 54 genes were shared between DEGs and the host genes of DETs. Notably, 219 genes exhibited significant differential expression at the transcript level despite showing no significant changes in overall gene expression (**Supplementary Figure 2A**). Similarly, in the Remission group, 157 genes were common to both DEGs and DET host genes, while 101 genes displayed transcript-level differences exclusively (**Supplementary Figure 2B**).

**Figure 3:**
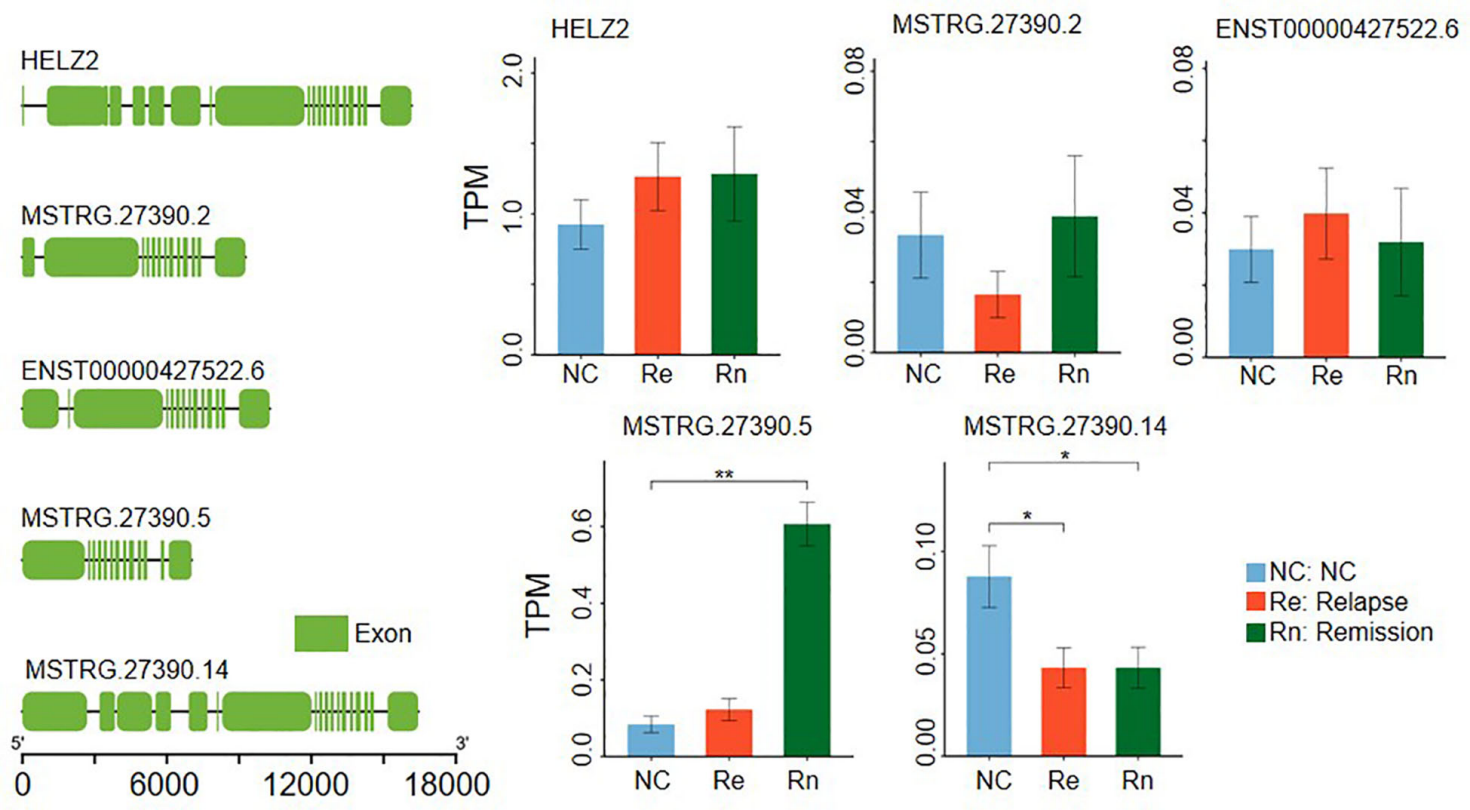
Comparative analysis of expression patterns between genes and transcripts. Genomic architecture of the *HELZ2* gene along with its four transcript isoforms. The bar charts display the respective expression levels of the *HELZ2* gene and its specific isoforms across the NC, Relapse, and Remission groups. The bar height represents the mean, and the error bars represent the standard error. Independent samples t-tests (student’s t-test) were used to compare the differences between the relapse group, the remission group, and the normal control group. “ns” indicates no significant difference, “*” indicates a statistically significant difference (P < 0.05), and “**” indicates a statistically significant difference (P < 0.01).

A representative example is *HELZ2*, which comprises 49 exons and showed no significant differential expression at the gene level in either the Relapse vs. NC or Remission vs. NC comparisons (**Figure 3**). However, its isoforms displayed distinct regulatory patterns. The transcript MSTRG.27390.2 (containing 13 exons) was significantly down-regulated in the Relapse group but showed no difference in the Remission group. Another isoform, ENST00000427522.6 (14 exons), remained stable across all groups. Furthermore, MSTRG.27390.5 (13 exons) was specifically up-regulated in the Remission group, while MSTRG.27390.14 (18 exons) was down-regulated in both the Relapse and Remission phases. These observations, where transcript-level shifts occur independently of total gene abundance, were prevalent in both disease stages. These findings suggest that alternative splicing may serve as a crucial regulatory mechanism, independent of gene expression abundance, contributing to the clinical progression and therapeutic response in GD.

### 3.4 Identification and characterization of AASEs

**Figure 4:**
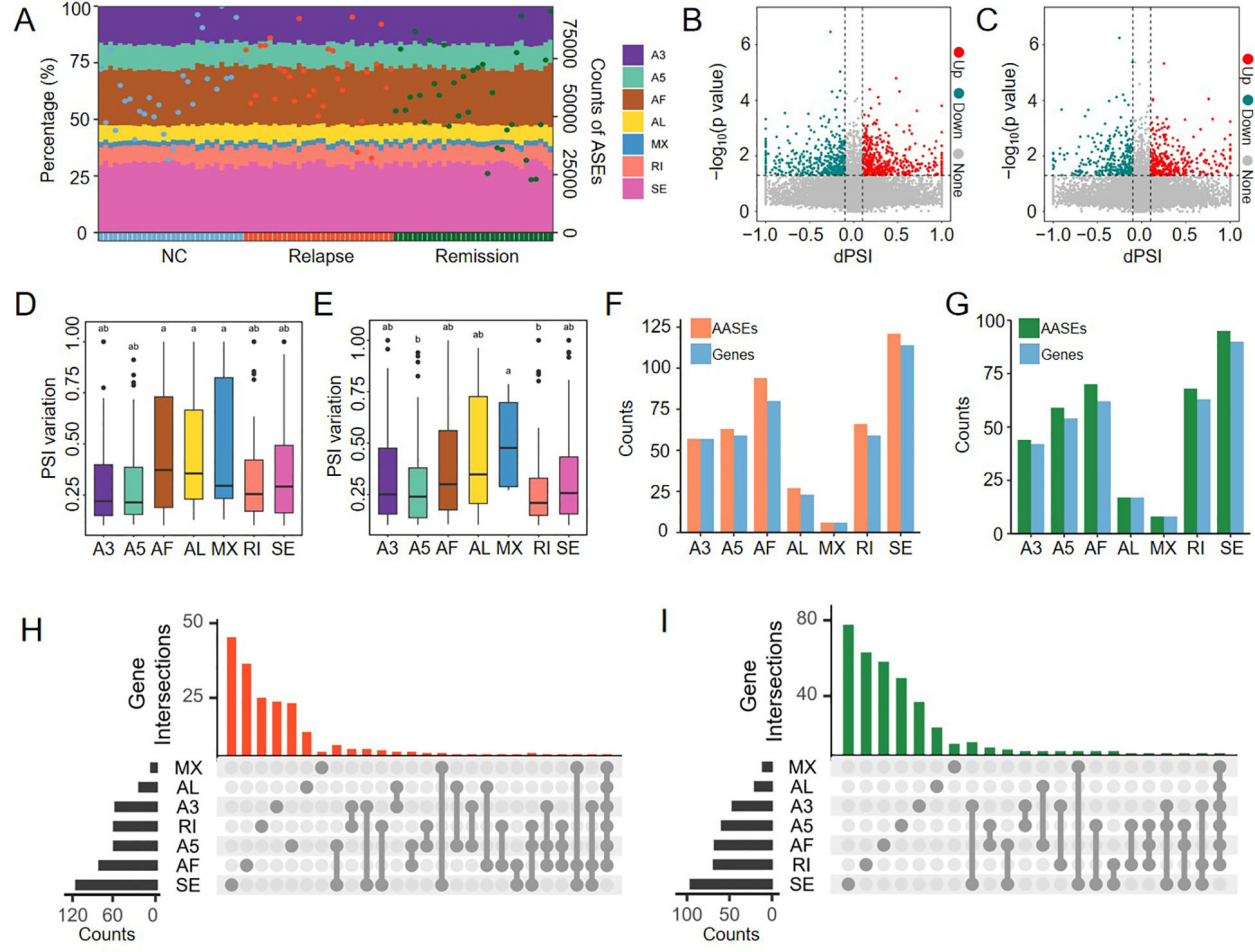
Characterization and differential analysis of AASEs in GD. **(A)** Stacked bar chart showing the proportions of different ASE types across 94 samples (left y-axis). Dots represent the total number of ASEs in each sample (right y-axis), with blue, red, and green dots indicating the NC, Relapse, and Remission groups, respectively. **(B, C)** Volcano plots illustrating AASEs in the Relapse vs. NC **(B)** and Remission vs. NC **(C)** comparison groups (p-value < 0.05 and |Delta PSI| > 0.1). Red and blue dots represent AASEs with significantly up-regulated and down-regulated PSI values in the Relapse or Remission groups, respectively. **(D, E)** Box plots displaying the distribution of |Delta PSI| values across the seven AASE types in the Relapse vs. NC **(D)** and Remission vs. NC **(E)** comparisons. Statistical significance was determined using one-way ANOVA followed by LSD *post-hoc* tests. Different letters indicate significant differences at the *P* < 0.05 level. **(F, G)** Bar charts showing the number of events and associated genes for the seven AASE types in the Relapse vs. NC **(F)** and Remission vs. NC **(G)** groups. **(H, I)** UpSet plots illustrating the number of genes associated with different AASE types or their combinations in the Relapse vs. NC **(H)** and Remission vs. NC **(I)** groups.

Given the pivotal role of alternative splicing in human pathogenesis (49), we systematically characterized the dysregulation of ASEs during the progression of GD across the Relapse and Remission phases. A total of 108,291 ASEs were identified, with the number of events per sample ranging from 22,804 to 97,622. These ASEs were categorized into seven distinct types: alternative 3’ splice site (A3), alternative 5’ splice site (A5), alternative first exon (AF), alternative last exon (AL), mutually exclusive exons (MX), retained intron (RI), and skipping exon (SE). The cumulative distribution of these seven ASE types remained remarkably consistent across the NC, Relapse, and Remission groups (**Figure 4A**). This stability suggests that the development of GD does not induce a global shift in the proportions of splicing types but rather involves the aberrant regulation of specific genes or splicing events.

To evaluate splicing variations relative to the healthy state, we identified AASEs in the Relapse and Remission groups compared to the NC group. In the Relapse vs. NC comparison, 858 AASEs were identified, consisting of 434 up-regulated and 424 down-regulated events (**Figure 4B**). In the Remission vs. NC comparison, 670 AASEs were detected, including 361 up-regulated and 309 down-regulated events (**Figure 4C**). Analysis of PSI variation demonstrated that the |Delta PSI| values for AF, AL, and MX were consistently higher than those of other AASE types in both the Relapse and Remission groups, whereas A5 and RI exhibited the smallest |Delta PSI| values (**Figures 4D, E**). Furthermore, the abundance of AASEs and their associated genes varied across types. SE was the most prevalent AASE type, with 121 and 95 events identified in Relapse and Remission, involving 114 and 90 genes, respectively. Conversely, MX was the least frequent type, with only 6 and 8 events involving 6 and 8 genes in the two respective groups (**Figures 4F, G**). Notably, we observed that the majority of affected genes in both Relapse and Remission were regulated by a single AASE type (**Figures 4H, I**). For instance, among the genes affected by AF-type AASEs, 70 out of 80 in Relapse and 56 out of 62 in Remission were exclusively regulated by AF, including key genes such as *STAT6*, *BCL6*, and *TAP1*. However, a small subset of genes was subject to multi-type AASE regulation. In the Relapse group, four genes, including *COPA*, were co-regulated by SE and RI, while *PLCD1* was simultaneously affected by RI, AF, and A5. These findings demonstrate that genes associated with AASEs in GD are influenced either by individual splicing events or combinations of different types, highlighting the intricate complexity of splicing-mediated transcriptional dynamics in GD.

### 3.5 Correlation network of RBPs and AASEs in GD

**Figure 5:**
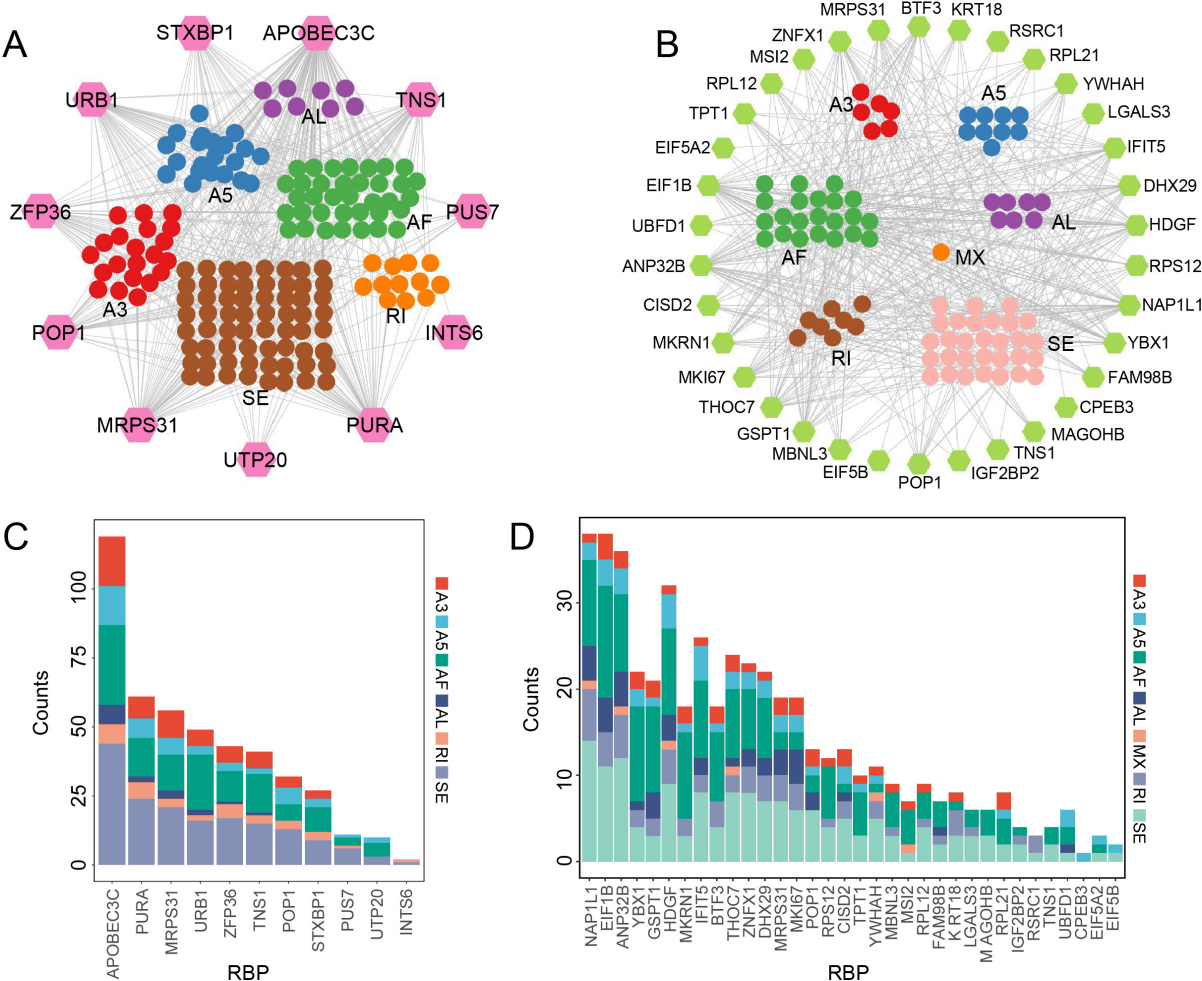
Correlation analysis of RBPs and AASEs in GD during the Relapse and Remission phases. **(A, B)** Splicing relevance networks of RBP-AASE associations in the Relapse group **(A)** and the Remission group **(B)** (|SCC| > 0.5 and p.adjust < 0.05). In the network nodes, the outer hexagons and inner circles represent RBPs and AASEs, respectively, while the edges indicate significant correlations between them. **(C, D)** Stacked bar charts illustrating the distribution of different AASE types associated by each RBP in the Relapse group **(C)** and the Remission group **(D)** networks. The x-axis displays the RBP genes, and the y-axis represents the number of associated AASEs.

To investigate the potential associations between RBPs and splicing variations, we performed Spearman correlation analysis between differentially expressed RBPs and AASEs. We identified 11 and 34 differentially expressed RBPs in the Relapse vs. NC and Remission vs. NC groups, respectively (**Supplementary Table 3**). In the Relapse vs. NC comparison, 451 RBP-AASE pairs exhibited significant correlations (|SCC| > 0.5 and p.adjust < 0.05), involving 11 RBPs and 162 AASEs (**Supplementary Table 4**). In the Remission vs. NC comparison, 34 RBPs were found to significantly correlate with 83 AASEs, forming 498 significant RBP-AASE pairs (**Supplementary Table 5**). Based on these associations, we constructed stage-specific RBP-AASE splicing relevance networks for the Relapse and Remission phases (**Figures 5A, B**). In addition, there were notable differences in the extent of associations among different RBPs. In the Relapse-associated network, *APOBEC3C* showed the highest number of correlated AASEs, being associated with 119 events across six distinct splicing types. In contrast, *INTS6* exhibited the fewest associations, with only two AASEs, while the remaining RBPs were associated with 10 to 61 AASEs each (**Figure 5C**). In the Remission group, *NAP1L1*, *EIF1B*, *ANP32B*, and *HDGF* displayed the highest numbers of associated AASEs, with 38, 38, 36, and 32 events, respectively (**Figure 5D**). These findings suggest that these RBPs may be more extensively associated with splicing variations characteristic of the GD remission phase following treatment.

### 3.6 Identification of sQTLs in GD

**Figure 6:**
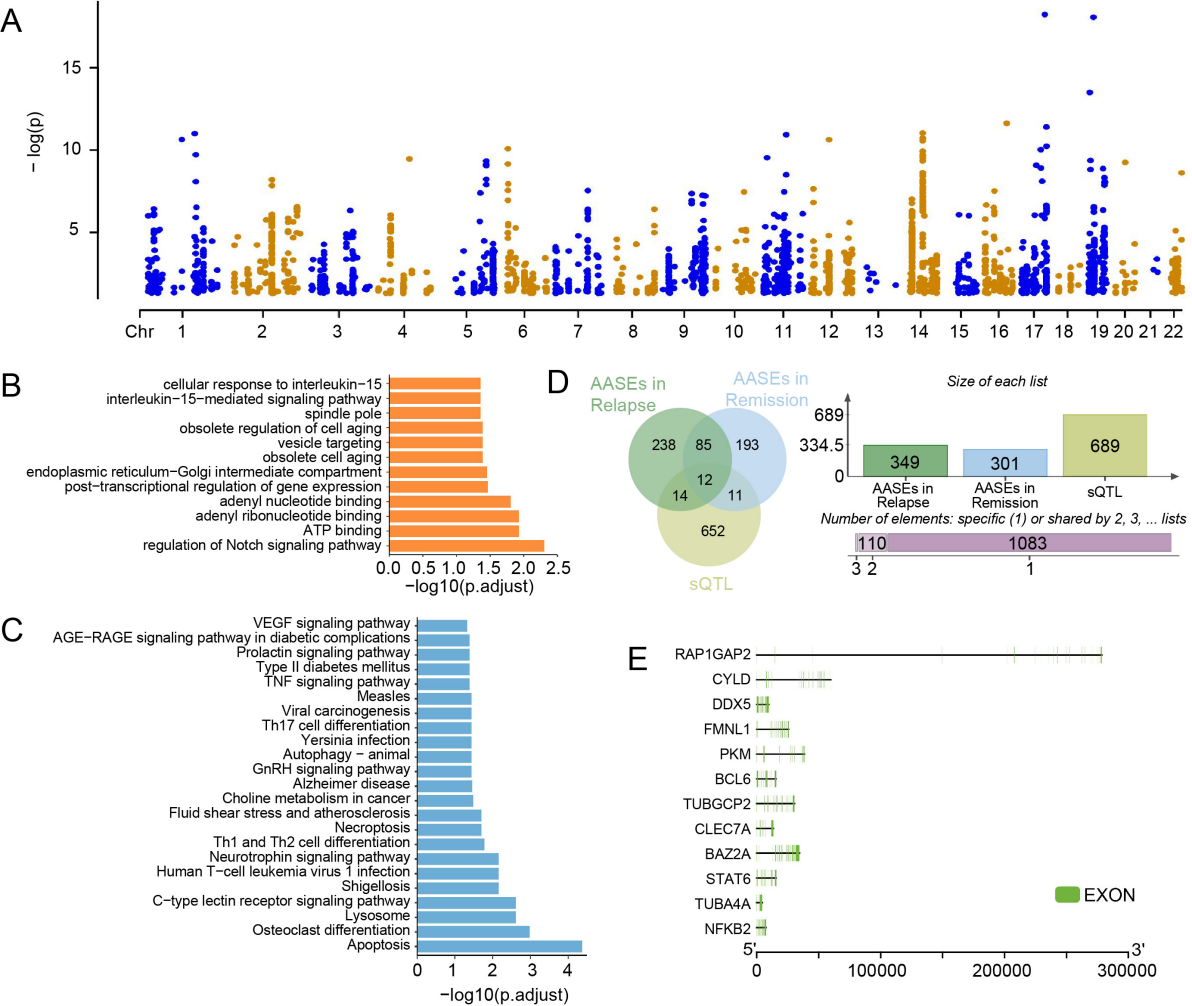
sQTL analysis and identification of candidate genes in GD. **(A)** Manhattan plot displaying the significant sQTLs identified across the genome in GD samples (p.adjust < 0.05). **(B)** Bar chart illustrating the GO enrichment results for the sGenes (p.adjust < 0.05). **(C)** Bar chart illustrating the KEGG pathway enrichment results for the sGenes (p.adjust < 0.05). **(D)** Venn diagram showing the overlap between sGenes and AASEs related genes in both the Relapse and Remission groups. **(E)** Identification of 12 sGenes that undergo aberrant alternative splicing during GD progression.

To systematically delineate the genetic regulatory landscape of alternative splicing in GD, we performed a comprehensive sQTL analysis by integrating 903,976 SNPs identified via RNA-seq with a PSI matrix encompassing 108,291 ASEs. Our analysis identified 4,507 significant sQTL-ASE associations (**Supplementary Table 6**), involving 2,848 unique sQTL SNPs and 1,084 ASEs (**Figure 6A**). These sQTL-regulated ASEs were associated with 689 genes, providing a direct link between genetic variation and splicing regulation in GD. Among them, these genes have an overlap gene (*FBXL19*) with the East Asian GD GWAS results from the GWAS Catalog (GCST90018627) (50), suggesting a possible link between genetic variation and splicing regulation in GD.

To elucidate the biological significance of these sGenes, we conducted GO and KEGG enrichment analysis. GO results revealed significant enrichment in the regulation of the Notch signaling pathway, ATP binding, and adenyl nucleotide binding (**Figure 6B**). These processes are pivotal for governing immune cell differentiation and maintaining cellular energy homeostasis, suggesting that sQTL-mediated splicing may influence GD pathogenesis by modulating Notch signaling and adenylate-related functions. Furthermore, KEGG enrichment analysis highlighted pathways closely linked to immune dysregulation, inflammatory responses, and programmed cell death, including Th1, Th2, and Th17 cell differentiation, the TNF signaling pathway, C-type lectin receptor signaling, necroptosis, and apoptosis (**Figure 6C**). These findings imply that genetic variants may drive the pathological progression of GD through the orchestration of immune imbalance and inflammation-associated cell death.

Notably, we found that among the 689 sGenes, 12 exhibited aberrant alternative splicing in both the Relapse and Remission stages (**Figure 6D**). These genes, including *BAZ2A*, *BCL6*, *CLEC7A*, *CYLD*, *DDX5*, *FMNL1*, *NFKB2*, *PKM*, *RAP1GAP2*, *STAT6*, *TUBA4A*, and *TUBGCP2*, likely represent critical molecular hubs where genetic variations manifest as splicing abnormalities to disrupt key immune signaling pathways throughout the GD clinical course. Detailed investigation into the genomic architecture and expression profiles of these candidates revealed that *DDX5* and *PKM* were significantly up-regulated in both Relapse and Remission phases compared to NC (**Figure 6E**; **Supplementary Figure 3**), suggesting that *DDX5* and *PKM* likely play pivotal roles in the Relapse and Remission phases of GD.

## 4 Discussion

GD constitutes a prototypical autoimmune disorder characterized by the breakdown of immune tolerance and the persistent activation of effector immune responses, driven by a complex interplay of genetic susceptibility and environmental triggers. While the immunological hallmarks of GD have been extensively characterized, the specific transcriptomic nuances distinguishing clinical relapse from remission, particularly at the resolution of transcript isoforms and AS regulation, remain largely elusive. By integrating gene and transcript-level expression profiling with systematic AASE identification and sQTL mapping, this study provides a comprehensive landscape of the stage-specific regulatory dynamics in GD. Importantly, while gene-level differential expression patterns observed in this study are largely consistent with previously reported immune dysregulation in GD, our analysis further reveals transcript-level and splicing-associated regulatory features that have not been systematically characterized, particularly in distinguishing relapse from remission. Our findings reveal that the relapse and remission phases are not merely distinguished by quantitative variations in transcriptional dysregulation but represent qualitatively distinct regulatory programs involving immune function, metabolic control, and splicing orchestration. Notably, the significant alterations in AS and its genetic regulation suggest that genetic factors may modulate the expression forms and functions of immune-related genes via splicing processes, thereby actively participating in the transition between disease states.

At the gene level, we observed that the majority of DEGs remained upregulated in both relapse and remission phases, suggesting that the immune system in GD patients fails to fully revert to a healthy baseline even after treatment. In the relapse phase, the upregulated DEGs did not enrich into specific functional pathways but rather indicated a state of global immune disequilibrium. This scattered activation pattern may reflect a weakened innate immune barrier, as evidenced by the significant downregulation of genes associated with antimicrobial humoral responses and TNF-mediated innate regulation. Previous studies have highlighted that defects in innate immunity and dysregulated inflammatory signaling can diminish the constraints on adaptive immunity, thereby lowering the threshold for autoimmune reactivation (51–53). Conversely, the remission phase was characterized by the upregulation of processes involving metabolic homeostasis, hematological functions, and apoptosis regulation, alongside the downregulation of TNF production pathways. These signatures suggest a transitional state toward physiological recovery and the attenuation of systemic inflammation, consistent with reports that reduced TNF levels can mitigate inflammatory severity in various autoimmune contexts (54, 55). Collectively, these results offer a novel biological perspective where relapse resembles a state of compromised immune barriers, while remission represents a progressive restoration of metabolic and inflammatory homeostasis. Furthermore, we observed that the downregulated DEG enrichment results during relapse and remission were closely associated with tissue repair, vascular integrity, and immune-mediated inflammatory responses. The reduced activity of these pathways during relapse may indicate impaired tissue recovery and hemostasis regulation, potentially leading to persistent inflammatory imbalance. Notably, this synergistic inhibition of hemostasis and repair-related pathways has been rarely reported in previous transcriptomic studies of GD, suggesting a previously under-recognized aspect of disease relapse. This finding provides new insights into the biological differences between relapse and remission, highlighting the potential role of vascular and repair mechanisms in the progression of GD.

However, gene-level differential expression analysis alone fails to capture the full complexity of the GD transcriptome. We identified numerous genes whose total abundance remained stable while their constituent isoforms underwent significant switching. This phenomenon, where transcript-level shifts occur independently of total gene expression, mirrors observations in other complex conditions such as endometriosis (56) and various plant stress responses (57). A compelling example is *HELZ2*, an RNA helicase-like protein containing zinc-finger and ATP-dependent helicase domains (58). Although its total expression was stable, specific *HELZ2* isoforms displayed stage-specific dysregulation in our data. Given its known involvement in innate immune regulation and inflammatory signaling (59, 60), our findings suggest that *HELZ2* may exert its pathogenic effects through isoform-specific functions rather than overall dosage changes. This underscores the necessity of integrating transcript-level information to uncover molecular drivers that remain hidden in traditional gene-level analysis.

AASEs represent a pivotal layer of post-transcriptional control that directly influences cellular signaling and function (61). The dominance of SE events identified in this study highlights that exon skipping may act as a core mechanism in GD pathogenesis. Interestingly, the regulation of transcript structures in both relapse and remission phases was predominantly characterized by a “single-splicing-type dominant” mode. This streamlined regulatory pattern may facilitate the fine-tuned and stable functional modulation of critical immune genes. In contrast, genes subject to multi-type splicing regulation, such as *COPA* and *PLCD1*, may serve as high-plasticity hubs within the splicing network, facilitating complex transitions between immune states. Our RBP-AASE network analysis further demonstrates that these splicing patterns are driven by stage-specific RBP programs. The relapse phase was prominently associated with widespread regulation by RBPs such as *APOBEC3C*, whereas the remission phase was characterized by a core RBP network including *NAP1L1*, *EIF1B*, *ANP32B*, and *HDGF*. Notably, *NAP1L1* has been shown to play a crucial role in driving inflammatory responses and governing immune cell development in rheumatoid arthritis (62), while *ANP32B* is implicated in adaptive immune dysregulation (63). These findings suggest that the clinical progression of GD is accompanied by a systematic remodeling of the splicing landscape, selectively orchestrated by distinct RBP networks.

Furthermore, the integration of sQTL analysis provides a mechanistic link between genetic susceptibility and the GD phenotype. The enrichment of sGenes in Th1/Th2/Th17 differentiation and the Notch signaling pathway, both of which are central to immune homeostasis and T-cell fate, reinforces the concept that genetic variants reprogram the immune landscape via splicing. Among the identified candidates, *DDX5* and *PKM* emerged as the most critical molecular nodes because they uniquely satisfy three convergent criteria: they function as sGenes, exhibit aberrant alternative splicing patterns, and maintain significant upregulation across both relapse and remission phases. *DDX5*, a key RNA helicase, is known to promote Th17-related cytokine expression in inflammatory environments (64, 65), while *PKM* serves as a master regulator of immunometabolic reprogramming by enhancing glycolysis and amplifying NF-κB signaling (66, 67). The sustained upregulation of these genes suggests that *DDX5* and *PKM* may drive persistent immune activation and metabolic shifts, acting as key conduits through which genetic risk manifests as the clinical hallmarks of GD. These molecules, therefore, represent potential targets for therapeutic intervention and risk assessment.

Despite the strengths of our comprehensive multi-layered transcriptomic analyses, several limitations should be considered. First, this study is based on bulk RNA-seq data, which may mask cell–type–specific transcriptional and splicing heterogeneity within complex immune populations. Future studies incorporating single-cell transcriptomic approaches would provide higher-resolution insights into cell–type–specific regulatory dynamics. Second, although we identified extensive associations between RBPs and AASEs, these relationships are derived from correlation analyses and do not establish direct regulatory mechanisms. Experimental validation, such as RBP perturbation assays or CLIP-seq, will be necessary to confirm causal interactions. While these limitations highlight important avenues for future improvement and validation, our study remains valuable in advancing the understanding of GD relapse and remission. In summary, our research elucidates the complementary roles of gene- and transcript-level regulation in GD. By highlighting the influence of aberrant AS and its genetic control via sQTLs, this study reveals the influence of splicing regulation in disease evolution. These insights not only advance our understanding of the molecular mechanisms underlying GD relapse and remission but also provide a rich resource of potential stage-specific markers and therapeutic targets for the management of GD.

## Data Availability

The datasets presented in this study can be found in online repositories. The names of the repository/repositories and accession number(s) can be found in the article/**Supplementary Material**.

## Supplementary material

Supplementary Figure 1 The PCA plot shows the cluster distribution among the 94 samples. MA plots illustrating differential gene expression in Relapse vs. NC, Remission vs. NC, and Relapse vs. Remission comparison groups (padj < 0.05 and |log2FoldChange| ≥ 1). Red dots represent significantly up-regulated genes, green dots represent significantly down-regulated genes, and gray dots indicate genes with no significant change in expression.

Supplementary Figure 2 Venn diagrams illustrating the overlap between DEGs and the host genes of DETs in the Relapse vs. NC comparison **(A)** and the Remission vs. NC comparison **(B)**.

Supplementary Figure 3 The bar chart shows the expression levels of 12 overlapping host genes of sQTL and AASEs in different samples.

Supplementary Table 1 Information on peripheral blood samples from 94 individuals with Graves’ disease.

Supplementary Table 2 –1236 RNA-binding proteins (RBPs) identified through literature search and screening.

Supplementary Table 3 Differentially expressed RBP genes in the Relapse vs NC and Remission vs NC comparison groups.

Supplementary Table 4 –451 significantly correlated RBP-AASE pairs (SCC > 0.5 and FDR < 0.05) were found in the Relapse vs NC comparison group.

Supplementary Table 5 –498 significantly correlated RBP-AASE pairs (SCC > 0.5 and FDR < 0.05) were found in the Remission vs NC comparison group.

Supplementary Table 6 Based on sQTL analysis of 94 samples, 4507 significant sQTL-ASE association pairs were identified.
