# Supplementary figures and images for: Integrated RNA-seq and sQTL analysis reveal immune and splicing regulatory features underlying relapse and remission after treatment of Graves’ disease
Source: Front Endocrinol (Lausanne). 2026 May 13;17:1791850. doi: 10.3389/fendo.2026.1791850 (PMC13212118; doi:10.3389/fendo.2026.1791850)

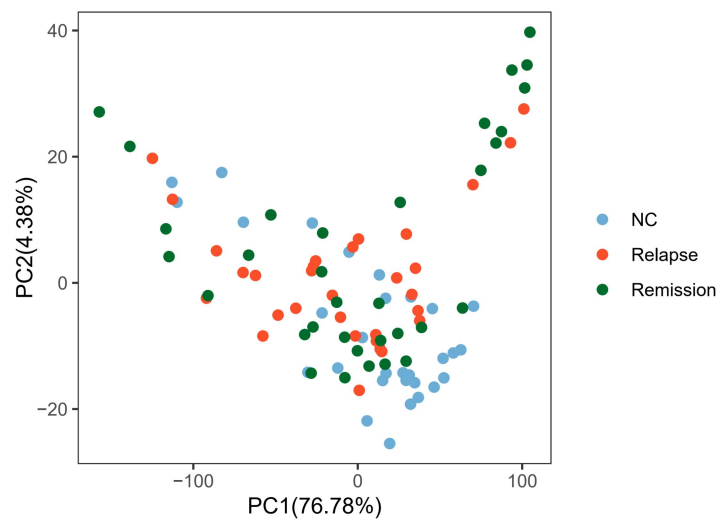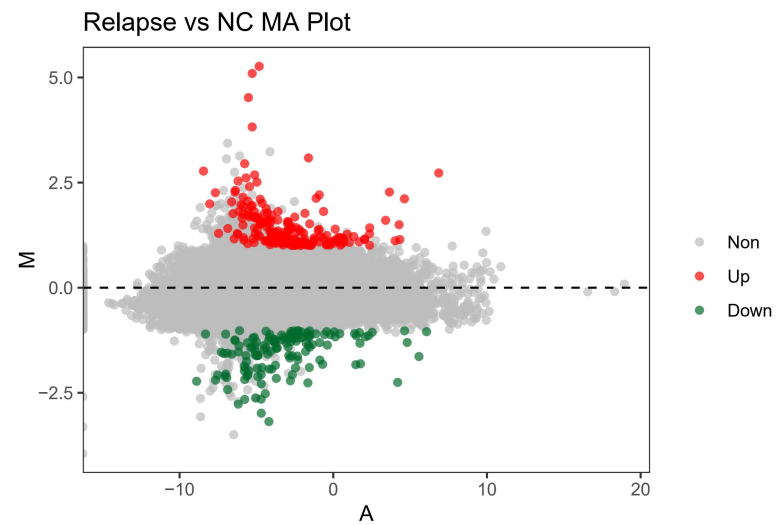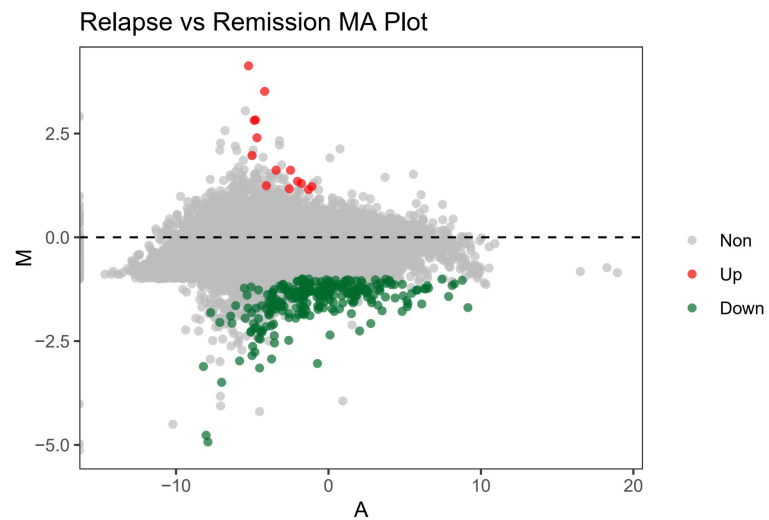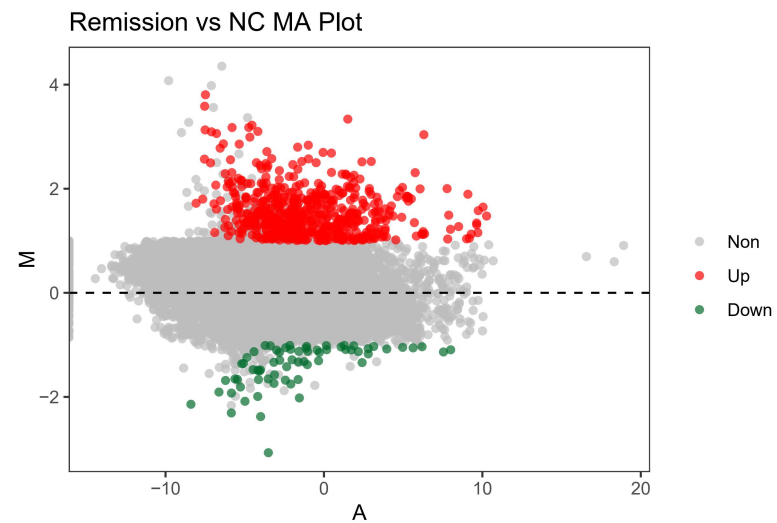

Supplement: Supplementary Figure 1 — The PCA plot shows the cluster distribution among the 94 samples. MA plots illustrating differential gene expression in Relapse vs. NC, Remission vs. NC, and Relapse vs. Remission comparison groups (padj < 0.05 and |log2FoldChange| ≥ 1). Red dots represent significantly up-regulated genes, green dots represent significantly down-regulated genes, and gray dots indicate genes with no significant change in expression. [file DataSheet1.pdf]

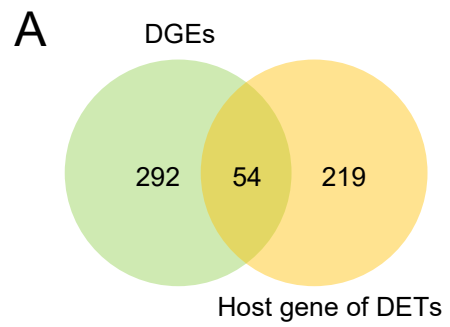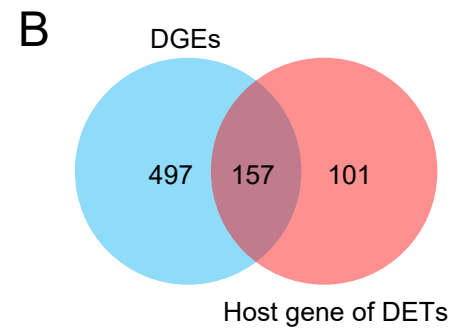

Supplement: Supplementary Figure 2 — Venn diagrams illustrating the overlap between DEGs and the host genes of DETs in the Relapse vs. NC comparison (A) and the Remission vs. NC comparison (B). [file DataSheet2.pdf]

EXON

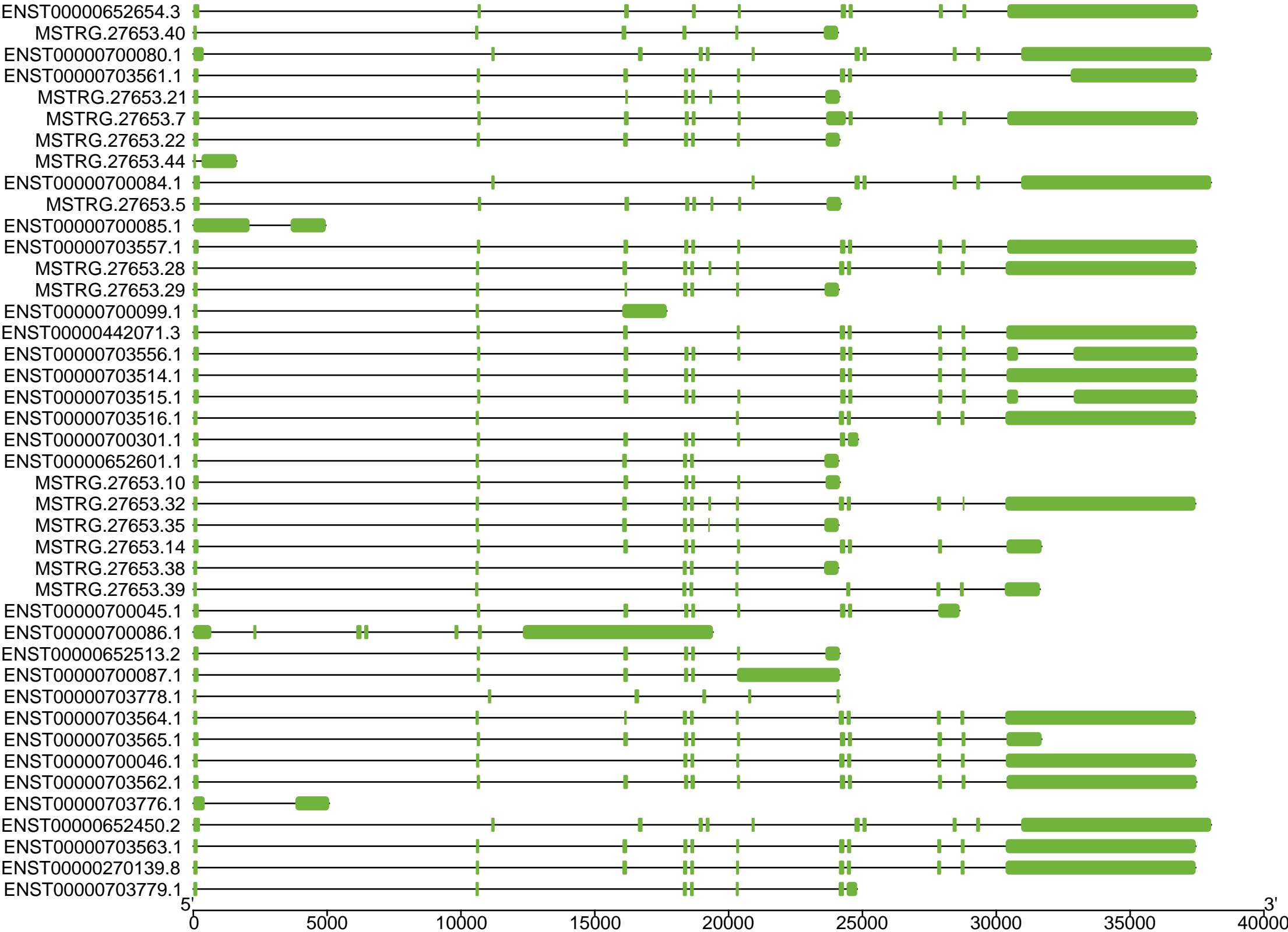

Supplement: Supplementary Figure 3 — The bar chart shows the expression levels of 12 overlapping host genes of sQTL and AASEs in different samples. [file DataSheet3.pdf]
